# Supplementary material for: Identification and functional analysis of non-coding regulatory small RNA FenSr3 in Bacillus amyloliquefaciens LPB-18
Source: PeerJ. 2023 May 15;11:e15236. doi: 10.7717/peerj.15236 (PMC10194069; doi:10.7717/peerj.15236)
Supplement: Supplemental Information 4 [file peerj-11-15236-s004.zip › KO/CK-vs-T1_map/map05161.html]

KEGG PATHWAY: Hepatitis B - Reference pathway


|  |  |
| --- | --- |
| **Hepatitis B - Reference pathway** |  |

[
Pathway menu
| Organism menu
| Pathway entry
| Show description
| User data mapping
]

Hepatitis B virus (HBV) is an enveloped virus and contains a partially double-stranded relaxed circular DNA (RC-DNA) genome. After entry into hepatocytes, HBV RC-DNA is transported to the nucleus and converted into a covalently closed circular molecule cccDNA. The cccDNA is the template for transcription of all viral RNAs including the pregenomic RNA (pgRNA), encoding for 7 viral proteins: large, middle, and small envelope proteins (LHBs, MHBs, and SHBs) that form the surface antigen (HBsAg), the core antigen (HBcAg), the e antigen (HBeAg), the HBV polymerase, and the regulatory protein X (HBx). The pgRNA interacts with the viral polymerase protein to initiate the encapsidation into the core particles. Through endoplasmic reticulum, the core particles finish assembling with the envelope proteins and are released. HBV infection leads to a wide spectrum of liver diseases raging from chronic hepatitis, cirrhosis to hepatocellular carcinoma. The mechanism of liver injury is still not clear. However, HBV proteins target host proteins, involved in a variety of functions, thus regulating transcription, cellular signaling cascades, proliferation, differentiation, and apoptosis.

|  |  |  |
| --- | --- | --- |
| Reference pathway | 184% 150% 122% 100% 82% 67% 55% | 图片下载 |
